# Longitudinal changes in DLPFC activation during childhood are related to decreased aggression following social rejection

**DOI:** 10.1073/pnas.1915124117

**Published:** 2020-03-31

**Authors:** Michelle Achterberg, Anna C. K. van Duijvenvoorde, Marinus H. van IJzendoorn, Marian J. Bakermans-Kranenburg, Eveline A. Crone

**Affiliations:** ^a^Leiden Consortium on Individual Development, Leiden University, 2333 AK Leiden, The Netherlands;; ^b^Institute of Psychology, Leiden University, 2333 AK Leiden, The Netherlands;; ^c^Leiden Institute for Brain and Cognition, Leiden University, 2333 ZA Leiden, The Netherlands;; ^d^Department of Psychology, Education and Child Studies, Erasmus University, 3062 PA Rotterdam, The Netherlands;; ^e^School of Clinical Medicine, University of Cambridge, Cambridge CB2 OSP, United Kingdom;; ^f^Department of Clinical Child and Family Studies, Vrije Universiteit Amsterdam, 1081 BT Amsterdam, The Netherlands

**Keywords:** social evaluation, childhood, brain development, social rejection, aggression regulation

## Abstract

Regulating aggression after social feedback is an important prerequisite for developing and maintaining social relations, especially in the current times with larger emphasis on online social evaluation. Studies in adults highlighted the role of the dorsolateral prefrontal cortex (DLPFC) in regulating aggression. Little is known about the development of aggression regulation following social feedback during childhood, while this is an important period for both brain maturation and social relations. The current study used a longitudinal design, with 456 twins undergoing two functional MRI sessions across the transition from middle (7 to 9 y) to late (9 to 11 y) childhood. Aggression regulation was studied using the Social Network Aggression Task. Behavioral aggression after social evaluation decreased over time, whereas activation in the insula, dorsomedial PFC and DLPFC increased over time. Brain–behavior analyses showed that increased DLPFC activation after negative feedback was associated with decreased aggression. Change analyses further revealed that children with larger increases in DLPFC activity from middle to late childhood showed stronger decreases in aggression over time. These findings provide insights into the development of social evaluation sensitivity and aggression control in childhood.

## Significance

Social rejection can result in negative self-evaluation, and individuals often display aggression for the purpose of self-protection. Regulating aggression after social evaluation in a socially adaptive way is an important prerequisite for establishing and maintaining social relationships. Here, we study the behavioral, neural, and genetic mechanisms of aggression regulation following social evaluation in middle childhood. This developmental phase marks important changes in both brain maturation and developing (online and offline) social relations. Our study showed that longitudinal changes in neural activation in the dorsolateral prefrontal cortex were associated with longitudinal changes in aggression regulation. These findings provide insights into how children experience social evaluation and how they control emotions following rejection.

Regulating emotions during social interactions is one of the most important requirements for developing social relationships in childhood. With increasing age, children become better at regulating their emotions (1), which has been suggested to be related to the development of cognitive and behavioral control functions between early childhood and adolescence (2, 3). In particular, receiving social evaluations such as acceptance and rejection can result in positive and negative self-evaluation (4). These social experiences can trigger control processes for the purpose of socially adaptive self-protection, such as controlling anger toward others (5, 6). Whereas adults have overall developed mechanisms to control behavioral responses to social evaluation (7), these mechanisms are still developing during adolescence (8).

Even though several studies have examined regulation processes in the context of social evaluation in adolescence, few studies have investigated the development of social emotion regulation during childhood, despite empirical findings showing that middle-to-late childhood marks the most rapid changes in cognitive control (9–11). Moreover, although neuroimaging studies have shed light on the underlying neurobiological changes that subserve childhood development in cognitive control, most studies have relied on cross-sectional comparisons, which hinders the possibility to examine within-person change. The current study builds upon new insights in the neural processing of social emotion regulation by examining change in neural and behavioral social control in a longitudinal functional MRI (fMRI) study in middle-to-late childhood. A second question concerns whether changes in behavior and neural activation are driven by genetic and/or environmental influences. This question was addressed by testing behavioral and neural change in a twin design including monozygotic (MZ) and dizygotic (DZ) twins (12).

**Fig. 1.**
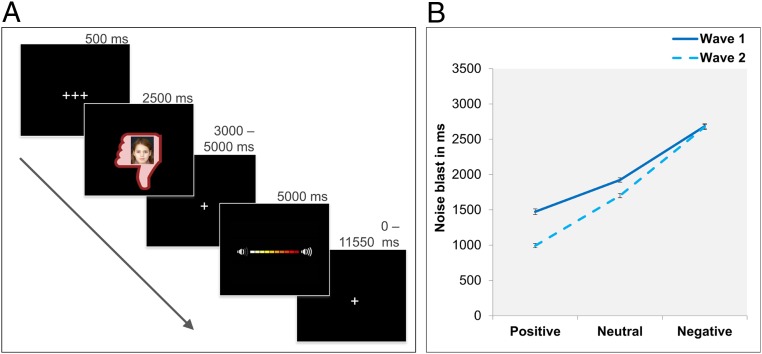
SNAT. (*A*) Visualization of one trial with negative social feedback. (*B*) Mean noise blast duration is influenced by condition (positive, neutral, negative), wave, and the interaction between condition and wave. Error bars represent SE of mean.

Neuroimaging research has shown that the significance of social evaluation is deeply rooted in our brain. Social evaluation, including social acceptance and rejection, has previously been studied using ecologically valid social judgment paradigms, in which participants’ profiles are evaluated by same-aged peers (13–16). Developmental neuroimaging studies including adolescent participants showed that receiving positive (acceptance) relative to negative (rejection) social feedback was associated with increased neural activity in the ventral medial prefrontal cortex (MPFC), the anterior insula (AI), and the anterior cingulate cortex (ACC) (17, 18). The Social Network Aggression Task (SNAT) (7) is an extended social evaluation paradigm that includes also a neutral-feedback condition and that provides participants with the opportunity to blast a loud noise toward the peer that evaluated them (Fig. 1*A*). Consistent with prior studies (19), it was found that both adults and children showed stronger ACC and AI activity in this task after receiving both positive and negative feedback (relative to neutral feedback), indicating that these regions signal social-salient cues (7, 20). Despite these findings for adults and adolescents, we currently have no knowledge of the neural responses to social evaluation feedback in childhood and their relation to behavioral aggression, even though social evaluation is already taking place in children. Nowadays, this topic is particularly relevant given that children are continuously connected to—and evaluated by—each other through multiplayer video gaming and social media (21).

It is well documented that social rejection can lead to aggression and retaliation (6, 22–26). Controlling emotions elicited by social evaluation feedback relies on cognitive control, that is, individuals with better cognitive control functions show less aggression following rejection, as signified by shorter noise blasts (22). Moreover, increased activation in the dorsal ACC and AI was related to less aggression after social rejection in adults with high executive functioning, whereas adults with low executive functioning showed increased aggression with increasing neural activation (22). Prior studies in adults further showed that the dorsolateral prefrontal cortex (DLPFC) might serve as a regulating mechanism for aggression after social evaluation, such that increased DLPFC activity after social rejection was related to less behavioral aggression (7, 27). Furthermore, stronger functional connectivity between the lateral PFC and limbic regions was related to less retaliatory aggression (25). Moreover, as the prefrontal cortex and executive functioning are still developing throughout childhood, children may be more sensitive to aggressive behavior after social rejection, as they might experience more difficulty with the regulation of social emotions (2, 28).

Interestingly, prior theoretical perspectives have suggested that DLPFC maturation is an important underlying mechanism for developing a variety of control functions in childhood (2, 28). Prior research revealed associations between DLPFC and behavioral aggression in 7- to 8-y-old children (20), although these were less pronounced than in adults (7). Taken together, studies in adults showed a link between cognitive control and regulation of emotions after rejection in the ACC/insula (22) and DLPFC (7), but no study to date has examined longitudinal developmental changes in these brain regions in childhood in the context of social evaluation. Moreover, the extent to which heritability and environmental factors contribute to these developmental changes is currently unknown.

The current study makes use of a developmental twin sample of the Leiden Consortium on Individual Development (L-CID) (29). This ongoing longitudinal twin study examines the development of social evaluation and behavioral control in 7- to 13-y-old children. The current study includes the first two fMRI assessments, separated by 2 y. A total of 492 same-sex twins (246 families) underwent two fMRI sessions across the transition from middle childhood (7 to 9 y) to late childhood (9 to 11 y). Using this unique study design, we address the following three research questions: 1) How does aggression regulation following social evaluation change longitudinally in middle to late childhood? 2) To what extent are behavioral changes related to (changes in) neural responses? 3) To what extent are changes in aggression regulation and associated neural responses explained by genetic and environmental influences?

**Fig. 2.**
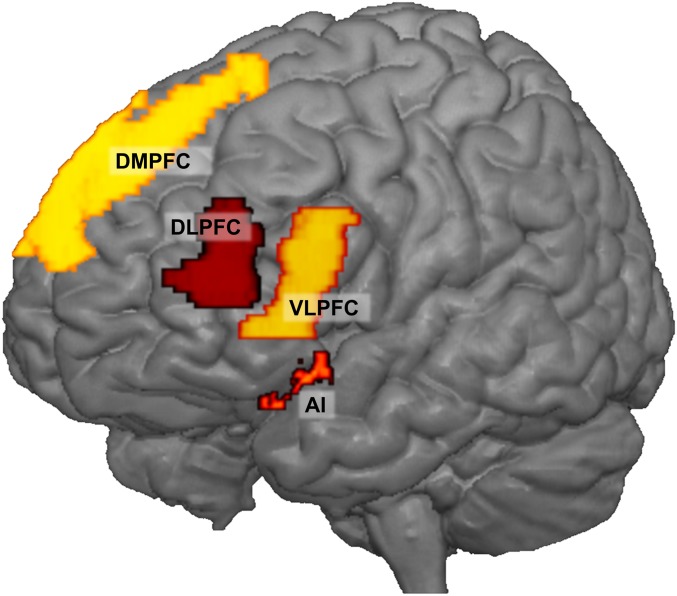
ROIs in the left hemisphere. The VLPFC and AI ROIs are bilateral; 3D nifti files of the ROIs are accessible through the OSF (https://osf.io/a4mdw/).

Using linear mixed-effects modeling, we first investigated how behavioral aggression after positive, negative, and neutral social feedback changed over time. Next, we investigated changes in brain responses related to positive, negative, and neutral social feedback longitudinally and examined brain–behavior associations. Based on previous studies (14, 20, 30), we selected the AI, the dorsomedial PFC (DMPFC), the ventrolateral PFC (VLPFC), and DLPFC as regions of interest (ROIs) (Fig. 2 and *SI Appendix*, Fig. S2). To examine individual differences in aggression regulation, we additionally performed exploratory brain–behavior MRI analyses to test for relations between brain activation and aggression regulation. These brain–behavior associations were followed by behavioral genetic modeling, using specific structural equation models based on twin similarities that provide estimates for heritability (31).

## Results

### Behavioral Aggression following Social Evaluation.

To test whether behavioral aggression decreased with increasing age, we performed a linear mixed-effect model on noise blast duration after social feedback across two waves. The linear mixed effect model for noise blast duration showed the expected main effect of type of social feedback (*SI Appendix*, Table S6). Noise blast duration was longer after negative feedback compared with neutral feedback, and shortest after positive feedback (all pairwise comparisons, *P* < 0.001). We also found the expected main effect of wave (*SI Appendix*, Table S6), with shorter noise blast durations at wave 2 (W2) compared with wave 1 (W1), indicating a decrease of behavioral aggression over time regardless of feedback valence. Follow-up analyses per condition showed that this decrease over time was significant for positive [*t*(2197) = 11.48, *P* < 0.001], neutral [*t*(2197) = 6.62, *P* < 0.001], and to a lesser extent also for negative social feedback [*t*(2197) = 3.56, *P* < 0.001]. Moreover, there was a significant condition × wave interaction effect (*SI Appendix*, Table S6). As can be seen in Fig. 1*B*, noise blast duration decreased more strongly between W1 and W2 after positive feedback than after negative feedback (*F* = 23.75, *P* < 0.001) and more after positive feedback than after neutral feedback (*F* = 16.27, *P* < 0.001). The same result was observed for neutral feedback: noise blast duration decreased more strongly between W1 and W2 after neutral feedback than after negative feedback (*F* = 5.00, *P* = 0.025). That is, over time, children showed a decrease in noise blast duration, and this effect was most pronounced for noise blasts following positive feedback (Fig. 1*B*).

### Confirmatory ROI Analyses.

Confirmatory ROI analyses were performed in two steps: first, we examined neural response patterns after social feedback across two time points in four ROIs (Fig. 2 and *SI Appendix*, Fig. S2) that were selected in a separate reference group (*Methods*). Second, we examined relations between changes in neural activity and noise blast durations.

#### Neural responses following social evaluation.

**Fig. 3.**
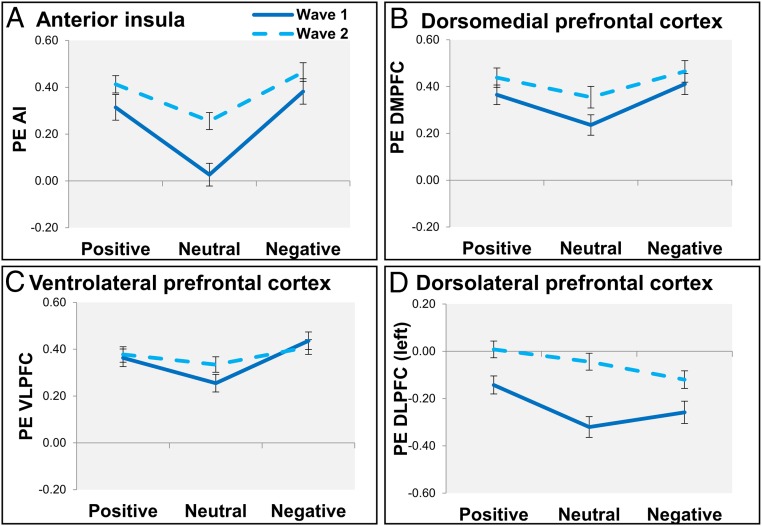
Neural activation after positive, neutral, and negative social feedback at W1 (solid dark blue lines) and W2 (dotted light blue lines) for the AI (*A*), the DMPFC (*B*), the VLPFC (*C*), and the left DLPFC (*D*).

To test for developmental changes in neural responses to social feedback, we performed linear mixed-effect models on four ROIs (AI, DMPFC, VLPFC, and DLPFC). We observed significant main effects of type of social feedback on neural activation in all ROIs (*SI Appendix*, Table S7). Patterns of activity differed between the ROIs. For the AI, DMPFC, and VLPFC, there was significantly more neural activation after negative and positive feedback relative to neutral feedback (Fig. 3 *A*–*C*), but the differences between positive and negative social feedback were not significant. For the DLPFC, in contrast, there was more activation after positive social feedback compared with both neutral and negative feedback but no significant difference between neutral and negative social feedback (Fig. 3*D*). Next, we addressed whether these activity patterns changed over time. We observed a significant effect of wave in the AI, the DMPFC, and the DLPFC, with generally stronger neural activation at W2 compared with W1 (Fig. 3 and *SI Appendix*, Table S7). There were no significant condition × wave interaction effects in ROI activation.

#### Neural responses following social evaluation and behavioral aggression.

To investigate brain–behavior associations in ROI activation, we added noise blast duration as a factor to the previously tested models. We found a significant main effect of noise blast duration in response to social feedback on AI and DLPFC activation (*SI Appendix*, Table S8). These findings indicated that increased AI activation was associated with longer noise blast after social feedback, regardless of valence (*B* = 1.11*e*^−4^), whereas increased DLPFC activation was associated with shorter noise blast after social feedback, regardless of valence (*B* = −3.57*e*^−5^). The DMPFC and VLPFC did not show significant brain–behavior associations.

### Exploratory Analyses.

Exploratory analyses were also performed in two steps: first, we conducted a whole-brain regression analysis including all available MRI data at W2 (*n* = 360). Next, we used the significant clusters as ROI to extract parameter estimates (PEs) per condition from MRI data at both waves, to explore brain–behavior associations across time.

#### Brain–behavior analyses on aggression following negative feedback.

**Fig. 4.**
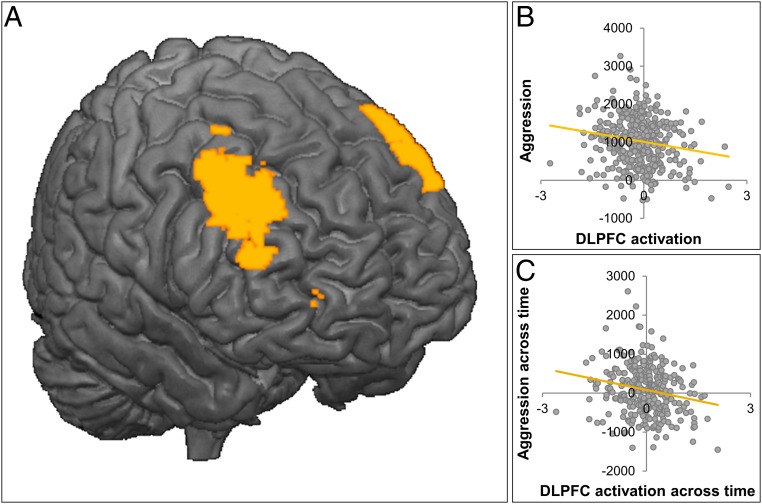
Whole brain–behavior analyses with all available MRI data at W2 (*n* = 360). (*A*) Significant cluster of activation in bilateral DLPFC for negative > neutral social feedback with noise blast (Δ negative–neutral) as regressor. (*B*) Visualization of brain–behavior association at W2: increased DLPFC activity after negative feedback is related to decreased aggression. (*C*) Brain–behavior association over time: the residualized change in DLPFC activation is negatively correlated to the residualized change in aggression, with larger increases in DLPFC activity over time being related to larger decreases in aggression.

We conducted a whole-brain regression analysis at W2 for receiving negative feedback (negative vs. neutral), with the difference in noise blast duration after negative and neutral feedback as a regressor (Δ Noise NegNeut W2; *Methods*). Consistent with our hypothesis, we observed a negative association between behavioral aggression and activation in the bilateral DLPFC (Fig. 4*A* and Table 1). Visualization of the effect (Fig. 4*B*) showed that an increase in DLPFC activation after negative feedback (relative to neutral feedback) was associated with less subsequent behavioral aggression. The unthresholded statistical map of this contrast is available through the NeuroVault (32) repository under https://neurovault.org/images/306500.

**Table 1.** MNI coordinates for local maxima activated for the whole brain–behavior contrast (negative feedback > neutral feedback with noise blast regressor [Δ negative–neutral]) in the whole sample (*n* = 360)

Anatomical region	Voxels	*P*_FWEcc_	T	*x*	*y*	*z*
Left dorsolateral prefrontal cortex	293	0.009	4.46	−36	17	55
			3.89	−48	26	40
			3.54	−48	14	43
Right dorsolateral prefrontal cortex	472	0.001	4.20	42	32	40
			4.02	48	41	31
			3.23	24	26	55

Results were FWE cluster-corrected (*P*_FWEcc_ < 0.05), with a primary voxel-wise threshold of *P* < 0.005.

To test for the specificity of the DLPFC activation, we conducted additional whole-brain regression analyses for the contrasts positive vs. neutral feedback (noise blast positive–neutral as regressor), positive feedback vs. fixation (noise blast positive as regressor), and neutral feedback vs. fixation (noise blast neutral as regressor). These analyses did not result in significant whole-brain activation. Moreover, multiple correlation analyses showed that the DLPFC activation from the whole-brain regression analyses (Fig. 4*A*) was significantly associated with the difference in noise blast duration between negative and positive feedback and after negative feedback (Table 2). There was no significant association between DLPFC activation and noise blast duration after positive feedback, neutral feedback, or the difference between positive and neutral feedback (all *P* values, >0.05; Table 2). These follow-up analyses indicate that the reported DLPFC activation is specific to negative social feedback and not to social feedback in general.

**Table 2.** Correlation analyses between DLPFC activity and noise blast duration to test for the specificity of DLPFC activation after negative social feedback

		Negative–positive noise blast	Negative noise blast	Positive–neutral noise blast	Positive noise blast	Neutral noise blast
DLPFC*	*r*	−0.18	−0.19	0.09	0.04	−0.04
	*P*	<0.001	<0.001	0.060	0.528	0.530

The correlations were controlled for the nestedness of the data by using HCSE estimators.

* Significant cluster from the whole-brain regression analyses with the difference in noise blast negative–neutral as regressor.

### Brain-Behavior Association Across Time.

To test whether children who showed larger increases in DLPFC activity over time also showed less behavioral aggression over time, we calculated the correlation between the residualized change scores for behavioral aggression and the residualized change scores for DLPFC activity (*Methods*). Note that for this analysis, we only included participants who had behavioral and brain data available at two waves (*n* = 293). For these participants, we calculated the relation between the residualized change in DLPFC activation (DLPFC activation at W2 corrected for DLPFC activation at W1; *Methods*) in whole-brain DLPFC ROI (Fig. 4*A*) and the residualized change in noise blast duration (noise blast at W2 corrected for noise blast at W1; *Methods*). We found a significant negative association (*r =* −0.22, *P* < 0.001; heteroscedasticity-consistent SE [HCSE]-corrected), indicating that children who showed the largest increase in DLPFC activation across childhood also showed the largest decrease in behavioral aggression in response to negative feedback across childhood (Fig. 4*C*).

Even though the sample was selected to represent a single age cohort, it is still possible that small age differences at the first assessment affected the results independent of longitudinal age changes. To explore the effect of age at W1 on the brain–behavior association across time, we computed a follow-up multiple-regression analysis with the residualized change in behavioral aggression as a dependent variable and the residualized change in DLPFC activation and age (at W1) as independent variables. The results confirmed that the residualized change in DLPFC activation across time was significantly associated with the residualized change in aggression across time (β = −0.22, *P* < 0.001, HCSE-corrected), whereas starting age was not significantly associated with the change in aggression across time (β = −0.06, *P* = 0.307, HCSE-corrected). This implies that within the relatively narrow age range of our sample, the association between changes in DLPFC activity and changes in behavioral aggression across the 2-y period were more strongly influenced by change over time than by differences in age at the first assessment.

### Genetic and Environmental Influences on Brain and Behavior.

As a follow-up to the brain–behavior analyses, we examined genetic and environmental influences by calculating within-twin correlations for MZ and DZ twin pairs (Table 3 and *SI Appendix*, Table S9). Behavioral genetic analyses revealed that behavioral aggression at W1 and W2 were influenced by both genetic influences (W1: A = 10% [95% CI = 0 to 40%], W2: A = 22% [95% CI = 11 to 56%]), shared environmental influences (W1: C = 8% [95% CI = 0 to 32%]; W2: C = 17% [95% CI = 7 to 46%]), and unique environmental influences (W1: E = 82% [95% CI = 60 to 98%]; W2: E = 62% [95% CI = 39 to 100%]). Behavioral aggression across time (residualized change), however, was only influenced by genetic influences (A = 16% [95% CI = 0 to 33%]) and unique environmental factors (E = 84% [95% CI = 67 to 100%]), with no influence of shared environmental factors (Table 3).

**Table 3.** Behavioral genetic modeling of behavioral aggression (noise blast negative–neutral) at W1, W2, and change across time (residualized change scores)

		MZ	DZ	A	C	E
Noise blast W1^†^	*r*	0.19*	0.25*	0.10	0.08	0.82
	*n*	138	115	[0.00 to 0.40]	[0.00 to 0.32]	[0.60 to 0.98]
Noise blast W2	*r*	0.22*	−0.04	0.22	0.17	0.62
	*n*	123	104	[0.11 to 0.56]	[0.07 to 0.46]	[0.39 to 1.00]
Noise blast change	*R*	0.22*	−0.06	0.16	0.00	0.84
	*N*	121	102	[0.00 to 0.33]	[ 0.00 to 0.19]	[0.67 to 1.00]

Numbers in brackets are 95% CIs. *P < 0.05.

^†^ See also Achterberg et al. (ref. 20).

Due to motion-related exclusions, the sample that was available for the behavioral genetic modeling of the fMRI data were considerably smaller (*n* = 48) than for the behavioral data (*n* = 102). Therefore, genetic and environmental influences could not be estimated reliably for DLPFC activation changes (33). For completeness, we report the estimations in the *SI Appendix*, Table S9.

## Discussion

There is a great need to have a better understanding of the mechanisms that drive changes in emotion regulation during social interactions across childhood. This question is more urgent than ever, given that young individuals currently connect not only through personal interactions but also through online communication (21) and therefore are continuously exposed to social evaluations. Negative social evaluations can trigger aggression (22–26), and controlling such retaliatory behaviors is an important socially adaptive mechanism to develop and maintain social relations. The current study examined the neural correlates of aggression regulation in childhood in response to social acceptance and rejection. For this purpose, we made use of unique data from a large longitudinal fMRI study, which allowed us to examine the development of aggression regulation following social evaluation within individuals across two time points. Our findings provide interesting insights on how frontal brain development associates with social emotion regulation over time in this important developmental phase. Specifically, the current study revealed three main findings: 1) behavioral aggression after social evaluation decreased over time, and this decrease was most pronounced for behavioral responses after positive and neutral social feedback; 2) confirmatory ROI analyses showed that increased activity in AI was related to more aggression following social feedback (regardless of its valence), whereas increased activity in DLPFC was correlated with less aggression; and 3) bilateral DLPFC activity was correlated to less subsequent aggression following negative social feedback. Longitudinal comparisons confirmed that larger increases in DLPFC activity across childhood were related to larger decreases in behavioral aggression, in particular after negative social feedback.

The behavioral results confirmed our initial hypothesis that behavioral aggression decreases over time, consistent with prior reports on age related increases in behavioral control (2, 3). Interestingly, however, these reductions in aggression were most pronounced following positive and neutral feedback, suggesting that participants were more motivated to refrain from aggression toward liked others. These results might also reflect an age-related increase in motor control, that is, older children might be better able to modulate their button presses. Our finding that children refrained from aggression toward liked others fits well with research showing that the importance of being liked and accepted by others increases over the course of childhood and into adolescence (8, 34). Thus, with increasing age, children become more focused on refraining punishment toward people with whom they socially connect and they differentiate more between liked (individuals signaling social acceptance) and disliked (individuals signaling social rejection) others (35). Increased behavioral regulation may therefore be mostly reflected by increased motor and cognitive capacity to refrain from punishing peers who previously accepted them.

By using functional neuroimaging, we were able to address the neural correlates following social evaluation feedback across two time points. Consistent with prior reports (20), children activate the same network across two waves, with stronger activity in AI, DMPFC, and VLPFC after both positive and negative social feedback (relative to neutral feedback). These findings fit well with results from the adult literature, showing that neural activation in DMPFC, AI, and VLPFC is associated with social rejection (36, 37) and signaling social-salient events (19). The DLPFC, in contrast, was more active for positive than negative feedback, comparable to the behavioral results showing a stronger reduction over time in aggression following positive feedback. Interestingly, AI and DLPFC also showed opposite relations to aggression. Even though both regions increased in activation over time, stronger AI activity was associated with more behavioral aggression and stronger DLPFC activity was associated with less behavioral aggression. The AI results are comparable to previous findings in adults with low executive control functions, showing that for individuals with low executive control, AI activity and aggression were positively correlated (22). An interesting direction for future research will be to examine whether this relation is stronger in childhood than adolescence and adulthood, when executive control functions increase.

The positive relation between DLPFC activity and aggression regulation was confirmed in the exploratory whole-brain analyses. Bilateral DLPFC activity was the only neural predictor in a whole-brain regression analysis for aggression control following negative relative to neutral feedback. These findings fit well with two decades of research pinpointing the DLPFC as an important region for cognitive control development (10, 38, 39). The current study extends this finding to the novel domain of social interactions and demonstrates that the same “cool” regulatory control functions are also important for regulation “hot” emotions in negative social evaluation contexts (11, 40). Moreover, DLPFC activity also explains individual differences in emotion regulation following rejection. A change–change analysis confirmed that those children who showed the largest increase in DLPFC activity after negative social feedback also had the largest reductions in behavioral aggression following negative feedback. Interestingly, even though cross-sectional analyses demonstrate both genetic and environmental influences on aggression regulation (20), change scores were only related to genetic influences, and although the heritability effects were modest in size, this is consistent with prior studies showing genetic effects on aggression (34, 35).

In addition to providing insights in the development of social emotion regulation during childhood, the current study also provides meaningful methodological considerations for future research. First, in the current study, aggression was operationalized in terms of noise blast duration in response to negative, neutral, and positive social feedback. Our ROI findings showed associations between the DLPFC and noise blast duration irrespective of its valence, which may question the role of DLPFC in aggression regulation specifically, as oppose to behavioral control in general. However, our exploratory analyses also revealed that the DLPFC was associated with negative feedback, a social context in which aggression is typically observed. Moreover, specificity analyses showed that the neural activation in this DLPFC region was specific to negative social feedback. Future studies should build upon these findings by investigating commonalities and differences in neural and behavioral correlates of reactions to social feedback with diverging valences.

Secondly, the current study covered a relatively narrow age range (7 to 9 y and 9 to 11 y), which provides a detailed analysis of middle childhood-related changes. Analyses including age showed that—within the relatively narrow age-range of our sample—the association between behavioral aggression and cortical indices of cognitive control is subject to age-related change but not to age differences at the first assessment. It should be noted that our exploratory longitudinal brain–behavior analyses were based on residualized change scores. Although these change scores reflect within-individual differences across time, they may also eliminate valuable information about individual variability over time (41). Individual variability in aggressive behavior is better captured using mixed multilevel models (42, 43), as we did in our confirmatory analyses. Future research should aim to replicate our exploratory longitudinal brain–behavior associations using more accurate and advanced mixed linear models (preferably using at least three time points) (44) to allow for disentangling general developmental patterns from individual differences in growth trajectories (45).

Lastly, the reliability of fMRI, specifically task-based fMRI has been heavily debated in recent years (46, 47). The variability observed in fMRI signals and the poor test–retest reliability in developing populations is a big concern for the field of developmental neuroscience (48). In our study, we also found rather low test–retest reliability across waves, with several contrasts even showing intraclass coefficients (ICCs) lower than 0.10, which has been suggested as a minimum level for multilevel analyses (49). It should be noted that 2 y between the two assessment waves is fairly long, with myriad potential influences to stimulate developmental change. Low ICCs across this time could either reflect noise in the fMRI measurement but might also reflect individual variability over time (50). Our results suggest that the latter is at least partly true, as we found predictable associations between behavioral and neural reactions to our feedback paradigm as well as specific developmental patterns of these responses pointing at growing maturation of inhibitory control over aggression after negative feedback.

Taken together, this study set out to test longitudinal changes in neural systems underlying social evaluation and aggression regulation and their relation to behavioral outcomes. We found an increase in behavioral control across childhood, as behavioral aggression decreased over time. Moreover, DLPFC activation was related to a decrease in behavioral aggression. Notably, children with larger increases in DLPFC activity across 2 y displayed the largest decrease in behavioral aggression over time. These results contribute to our understanding of how the developing brain processes social feedback and suggest that the DLPFC might serve as emotion regulation mechanisms in the context of negative social feedback.

## Methods

### Participants and Procedure.

Participants in this study took part in the longitudinal twin study of the L-CID (29). The procedures were approved by the Dutch Central Committee for Human Research, and written informed consent was obtained from both parents; 512 children (256 families) between the ages 7 and 9 y were included at the first wave (previously described in refs. 20 and 51), with a mean age of 7.94 ± 0.67 y (49% boys, 55% MZ). The majority of the sample was of caucasian etnicity (91%) and right-handed (87%). Ten participants (2%) were diagnosed with an Axis I disorder: eight with attention-deficit hyperactivity disorder; one with generalized anxiety disorder, and one with pervasive developmental disorder- not otherwise specific (PDD-NOS). Intelligence quotient (IQ) was estimated at W1 with the subtests “similarities” and “block design” of the Wechsler Intelligence Scale for Children, third edition. Estimated IQs were in the normal range (72.50 to 137.50); 456 children participated in a second laboratory visit 2 y later (for details regarding participant dropout, see *SI Appendix*, Fig. S1). Table 4 provides an overview of demographic characteristics of the sample at W1 and W2.

**Table 4.** Demographic characteristics of complete sample at W1 and W2

	W1	W2
*N*	512	456
Boys	49%	48%
Left-handed	13%	12%
AXIS I disorder*	2.1%	1.8%
SES*^,^^†^ low–middle–high	9%–46%–45%	7%–46%–47%
Age (SD)	7.94 (0.67)	9.98 (0.69)
Age range	7.02 to 9.68	8.97 to 11.68
Mean IQ* (SD)	103.58 (11.76)	103.81 (11.63)
IQ range	72.50 to 137.50	72.50 to 137.50

* At W1.

^†^ Social economic status (SES), based on parental education.

Participants underwent an MRI scan as part of the laboratory visits. At W1, 385 participants were included in the MRI analyses (mean age, 7.99 ± 0.68 y; 47% boys; see also ref. 20). At W2, 360 participants were included in the MRI analyses (mean age, 10.01 ± 0.67 y; 48% boys). A total of 293 participants were included in the MRI analyses at both waves (mean age W1: 7.99 ± 0.66 y; 47% boys). In between the two sessions, 91 (37%) randomly selected families received a short-term intervention aimed at promoting parental sensitivity and positive limit-setting (for details, see ref. 29). Given that this was not the focus of the current study, we controlled for group status (intervention group or control group) in our analyses. Twenty-seven families did not comply with random assignment to the parental intervention. The MRI data of these participants were used to create task-relevant independent ROIs.

### SNAT.

The SNAT, as described by Achterberg and coworkers (7, 20), was used to measure aggression after social feedback regardless of its valence. Participants viewed pictures of peers that gave positive, neutral, or negative feedback to the participant’s profile. Following each peer feedback, the children were instructed to imagine that they could send a loud noise blast to this peer, the duration of which was used as an index of aggression. To keep task demands as similar as possible between the conditions, participants were instructed to always press the button. The longer they pressed the button, the more intense the noise would be, which was visually represented by an intensity bar (Fig. 1*A*). Each trial started with a fixation screen (500 ms), followed by the social feedback (2,500 ms). After another jittered fixation screen (3,000 to 5,000 ms), the noise screen with the intensity bar appeared, which was presented for a total of 5,000 ms. Children were instructed to deliver the noise blast by pressing one of the buttons on the button box attached to their legs, with their right index finger. As soon as the participant started the button press, the intensity bar started to fill up with a newly colored block appearing every 350 ms. After releasing the button, or at maximum intensity (after 3,500 ms), the intensity bar stopped increasing and stayed on the screen for the remainder of the 5,000 ms. Participants received instructions on how to perform the SNAT, and the children were exposed to the noise blast during a practice session. Participants did not hear the noise during the fMRI session, to prevent that pressing the button would punish the participants themselves. The SNAT consisted of 60 trials, 3 runs of 20 trials for each feedback condition (positive, neutral, negative). The optimal jitter timing and order of events were calculated with Optseq 2 (52). ICC analyses (modeled with a two-way mixed model using the consistency definition) showed low (ICC < 0.40) (53) consistency in noise blast duration after positive (ICC = 0.32 [95% CI = 0.24 to 0.41]), neutral (ICC = 0.26 [95% CI = 0.17 to 0.35]), and negative feedback (ICC = 0.17 [95% CI = 0.08 to 0.26]) between W1 and W2.

### MRI Data.

#### Acquisition.

MRI scans were acquired with a standard whole-head coil on a Philips Ingenia 3.0 Tesla MR system. To prevent head motion, foam inserts surrounded the children‘s’ heads (54). The SNAT was projected on a screen that was viewed through a mirror on the head coil. Functional scans were collected during three runs T2*-weighted echo planar images (EPIs). The first two volumes were discarded to allow for equilibration of T1 saturation effect. Volumes covered the whole brain with a field of view (FOV) = 220 (anterior–posterior [ap]) × 220 (right–left [rl]) × 111.65 (foot–head [fh]) mm; repetition time (TR) of 2.2 s; echo time (TE) = 30 ms; flip angle (FA) = 80°; sequential acquisition, 37 slices; and voxel size = 2.75 × 2.75 × 2.75 mm. Subsequently, a high-resolution three-dimensional (3D) T1 scan was obtained as anatomical reference (FOV= 224 [ap] × 177 [rl] × 168 [fh]; TR = 9.72 ms; TE = 4.95 ms; FA = 8°; 140 slices; voxel size = 0.875 × 0.875 × 0.875 mm]).

#### Preprocessing.

MRI data were analyzed with SPM8 (Wellcome Trust Centre for Neuroimaging). The exact same preprocessing steps were used in preprocessing MRI data from W1 and W2. Images were corrected for slice-timing acquisition and rigid-body motion. Functional volumes were normalized to subject-specific anatomical images (3D T1) using affine transform mapping. Next, the subject-specific anatomical image is normalized to a T1 Montreal Neurological Institute (MNI) template using a nonlinear warp. These warp parameters are then applied to the EPI, resulting in MNI-normalized EPI data (55). By including the subject’s T1 scan, we can base the nonlinear warp to MNI space on a higher spatial resolution image. However, for some individuals, we did not have a sufficient T1 scan (*n* = 5 at W1; *n* = 10 at W2). For these participants, we used normalization using an EPI MNI template, which allows spatial normalization without the requirement of a T1 scan (56). Volumes of all participants were resampled to 3 × 3 × 3 mm voxels. Data were spatially smoothed with a 6-mm full-width-at-half-maximum isotropic Gaussian kernel. Translational movement parameters were calculated for all participants. Participants with at least two out of three runs of fMRI data with <3 mm (one voxel) motion in all directions were included in subject-specific analyses (W1: *n* = 385; W2: *n* = 360).

#### Subject-specific analyses.

Statistical analyses were performed on individual subjects’ data using a general linear model, previously described in ref. 20. The fMRI time series were modeled as a series of two events convolved with the hemodynamic response function (HRF). The onset of social feedback was modeled as the first event, with a zero duration and with separate regressors for the positive, negative, and neutral peer feedback. The start of the noise blast was modeled as the second event, with the HRF modeled for the length of the noise blast and with separate regressors for noise blast after positive, negative, and neutral judgments. Trials on which the participants failed to respond in time were modeled separately as covariate of no interest and were excluded from further analyses. Additionally, six motion regressors (corresponding to the three translational and rotational directions) were included as covariates of no interest. The least-squares PEs of height of the best-fitting canonical HRF for each condition were used in pairwise contrasts. The pairwise comparisons resulted in subject-specific contrast images.

### Confirmatory ROI Analyses.

#### ROI selection.

ROIs were based on higher-level group analyses of W2 in a separate reference group (the nonrandomized control group, *n* = 41; *SI Appendix*, Table S1). The advantage of this approach is that the participants were in exactly the same study protocol but were not included in the subsequent analyses, leading to an independent selection of ROIs (57). Using comparable sample sizes, we previously reported replicable results of main effects of the SNAT (58). SPM8’s MarsBaR toolbox (59) was used to construct ROIs based on the whole brain contrast by masking significant activation with regions from the Automated Anatomical Labeling atlas (60). We first investigated social feedback (positive, neutral, negative) versus fixation (*SI Appendix*, Fig. S3*A* and Table S1). Based on a priori hypotheses, we selected the bilateral AI, VLPFC, and DMPFC. In addition to the all feedback vs. fixation contrast, we also investigated the specific conditions. From the contrast positive vs. negative social feedback (*SI Appendix*, Fig. S3*B* and Table S1), we selected the left DLPFC as an additional ROI (Fig. 2). The contrasts negative vs. positive social feedback did not result in clusters of significant activation. The contrasts positive vs. neutral social feedback and negative vs. neutral social feedback resulted in increased activation in occipital (visual) cortex (*SI Appendix*, Table S1), but given that this was not an a priori hypothesized area, this region was not included in ROI selection.

Thus, in total, four ROIs were used in further analyses: the bilateral AI, bilateral VLPFC, the DMPFC, and the left DLPFC (Fig. 2). Sagittal and axial visualization of the ROIs are provided in *SI Appendix*, Fig. S2; 3D nifti files of the ROIs are accessible through the Open Science Framework (OSF) (https://osf.io/a4mdw/). PEs (averaged beta values) were extracted from the subject-specific contrasts (positive vs. fixation, neutral vs. fixation, and negative vs. fixation) for the entire sample minus the reference group with available MRI data on W1 (*n* = 343) and W2 (*n* = 317). ICC analyses (two-way mixed model using consistency) showed low consistency (ICCs < 0.40) (53) in brain activation for the contrasts negative > neutral, negative > positive, and positive > neutral feedback between W1 and W2 (*SI Appendix*, Table S5).

#### Linear mixed-effects models.

To test time-related changes in participant’s behavior and ROI brain activation, we used linear mixed-effects models using the lme4 package (61) in R (62). For these analyses, we included the whole sample minus the reference group (*n* = 458). Data were fitted on the average noise blast duration (for behavior) and average PEs (for ROIs) after positive, neutral, and negative social feedback. Two random effects were included to account for the nesting of condition and waves within the participant (ChildID) and the nesting of twin pairs within families (FamilyID). Fixed effects included feedback condition (three levels: positive, neutral, and negative) and wave (two levels: W1 and W2), while controlling for intervention group (two levels: intervention and control). Sex and estimated IQ (grand mean-centered) were included as additional covariates and all main effects and two-way interactions between covariates and feedback condition were included (sex × condition and condition × IQ). The fitted mixed-effect model was specified in R as:

$$\begin{array}{l} Noise/ROI\;∼\;condition\;\times \;wave\;\times \;intervention\;+\;condition\;\times \;sex+\;condition\;\times \;IQ\;+\;\left(1|childID\right)\;+\;\left(1|familyID\right). \end{array}$$

In addition, we examined associations between brain and behavioral responses, in which we were specifically interested in the extent to which behavior was associated with neural activation. To this end, we added noise blast duration to the model including all two- and three-way interactions with condition and wave. Results were inspected with type III ANOVA’s using Satterthwaite’s method. Significant main effects of condition were further inspected using least-square means, with Kenward–Roger corrected degrees of freedom and Bonferroni-adjusted *P* values.

### Exploratory Analyses.

#### Brain–behavior analyses.

In addition to neural responses to social feedback, we also examined whole brain–behavior relations in late childhood (W2). Results were family-wise error (FWE) cluster-corrected (*P*_FWEcc_ < 0.05), with a primary voxel-wise threshold of *P* < 0.005 (uncorrected) (63). Similarly to previous brain–behavior analyses in adults (7) and middle childhood (20), we conducted a whole brain regression analysis at the moment of receiving negative social feedback (negative vs. neutral), with the difference in noise blast duration after negative and neutral feedback as a regressor. In this way, we tested how initial neural responses to feedback were related to subsequent aggression. The difference in noise blast was computed by:

ΔNoise NegNeut W2=Negative noise blast W2− Neutral noise blast W2.

#### Brain–behavior associations across time.

To investigate brain–behavior associations across time, we computed the development over time in noise blast duration for the contrast negative–neutral and for brain activation in this contrast by calculating residual scores. In doing so, we could investigate the association between brain and behavior at W2, while correcting for the level of brain activation and aggressive behavior at W1. First, we conducted multiple regression analyses where behavioral aggression at W2 (Δ Noise NegNeut W2) was predicted by behavioral aggression at W1 (Δ Noise NegNeut W1). We then used the unstandardized residuals of this prediction as indicators of the development of behavioral aggression across time. The same was done for brain activation: brain activation at W1 (Δ Brain NegNeut W1) was regressed on brain activation at W2 (Δ Brain NegNeut W2), and we used the unstandardized residuals as indicators of the development of brain activation across time. Next, we calculated the correlation between the residualized change in behavior and the residualized change in brain activation.

Due to the nested nature of twin data, the data violate the assumption of homoscedasticity. Although the estimator of the regression parameters is not influenced when this assumption is violated, the estimator of the covariance matrix can be biased, resulting in too liberal or too conservative significance tests. Therefore, we used HCSE estimators, by using the HCSE macro of Hayes and Cai (64), with the HC3 method (65). A total of 293 participants had behavioral and brain data available at two waves and were included in the analyses regarding brain–behavior associations over time.

#### Behavioral genetic analyses.

The sample consisted of an approximately equal number of MZ and DZ same-sex twins, which provides the unique opportunity to test whether change is associated with genetic or environmental influences. As a follow-up to the exploratory brain–behavior analyses, we therefore examined genetic and environmental influences on brain and behavior by calculating Pearson within-twin correlations for MZ and DZ twin pairs. Similarities among twin pairs can be due to additive genetic variance (A) and common (shared) environmental factors (C), while dissimilarities can be ascribed to unique environmental influences and measurement error (E). We used behavioral genetic modeling with the OpenMX package (31) in R (62) to calculate these A, C, and E estimates. Per convention, the correlation of the shared environment (factor C) was set to 1 for both MZ and DZ twins, while the correlation of the genetic factor (A) was set to 1 for MZ twins and to 0.5 for DZ twins. The last factor, unique environmental influences and measurement error, was freely estimated. High estimates of A indicate that genetic factors play an important role, while estimates for C indicate influences of the shared environment, making twins in the same family more similar. In case the E estimate is highest, variance is mostly accounted for by unique environmental factors (making twins in the same family more different) and measurement.

#### Sensitivity analyses on a genetically independent sample.

As both the ROI selection (*n* = 41) as well as the analysis including all available MRI data at W2 (*n* = 360) are based in twin samples, our results might be influenced by the nestedness of our data. We therefore conducted additional sensitivity analyses on a genetically independent sample. Of the 360 participants with available MRI data at W2, there were 42 “single” twins (i.e., their twin brother/sister was excluded due to motion) and 159 complete twin pairs. Of these complete pairs, we randomly selected either the oldest or youngest twin, resulting in a genetically independent sample of *n* = 201. Sensitivity analyses are described in detail in *SI Appendix*. The use of this genetically independent sample did not meaningfully change our findings.

### Data Availability.

The group-level MRI data are available in NeuroVault at https://neurovault.org/collections/6070. Anonymized single-subject behavioral and ROI data that support the findings of this study are available in DataverseNL at https://hdl.handle.net/10411/BE2X5L. The European General Data Protection Regulation (GDPR) prevents us from uploading raw MRI data as this could compromise the privacy of research participants. However, the raw MRI data that support the findings of this study are available on request from the corresponding author.

## Supplementary Material

Supplementary File
