# Supplementary material for: Longitudinal changes in DLPFC activation during childhood are related to decreased aggression following social rejection
Source: Proc Natl Acad Sci U S A. 2020 Mar 31;117(15):8602–10. doi: 10.1073/pnas.1915124117 (PMC7165424; doi:10.1073/pnas.1915124117)
Supplement: Supplementary File [file pnas.1915124117.sapp.pdf]

Longitudinal changes in DLPFC activation during childhood are related to decreased aggression following social rejection

Michelle Achterberg, MSc [1,2,3*], Anna C.K. van Duijvenvoorde, PhD[1,2,3], Marinus H. van IJzendoorn, PhD [1,4,5] Marian J. Bakermans-Kranenburg, PhD [1,3,6] & Eveline A. Crone, PhD [1,2,3]

Affiliations:

[1] Leiden Consortium on Individual Development, Leiden University, the Netherlands

[2] Institute of Psychology, Leiden University, the Netherlands

[3] Leiden Institute for Brain and Cognition, Leiden University, the Netherlands

[4] Department of Psychology, Education and Child Studies, Erasmus University, The Netherlands

[5] School of Clinical Medicine, University of Cambridge, UK

[6] Department of Clinical Child and Family Studies, VU Amsterdam, the Netherlands

SI APPENDIX

***Corresponding author**: Michelle Achterberg, Faculty of Social Sciences, Leiden University, Wassenaarseweg 52, 2333 AK Leiden, The Netherlands. Tel: +31 71 527 6861, E-mail: m.achterberg@fsw.leidenuniv.nl

www.pnas.org/cgi/doi/10.1073/pnas.1915124117

Supplementary materials

Participants and sample selection

Supplementary Table 1 provides a flow-chart of participant selection and exclusion. Of the initial 256 families, 10 families (3.8%) dropped out of the study directly after wave 1 (W1), whereas one family (n=2) was included in the L-CID study after W1. An additional 19 families (7.4%) dropped out before wave 2 (W2), after randomization of the parental intervention (see Figure S1). The remaining 456 children participated in a second lab visit at W2 (time between waves 2.06±0.10, time range: 1.86-2.53). Participants underwent an MRI scan as part of the lab visits. All anatomical MRI scans were reviewed and cleared by a radiologist from the radiology department of the Leiden University Medical Center (LUMC). Four anomalous findings were reported. To prevent registration errors due to anomalous brain anatomy, these participants were excluded. At W1, 27 participants did not start the scan due to anxiety (n=13), contraindications (n=6), or lack of parental consent for MRI participation (n=4), or technical issues with the MR system (n=4)[s1]. Eighty-nine participants were excluded at W1 due to excessive head motion, which was defined as >3 mm motion (1 voxel) in any direction (x, y, z) in more than 2 runs of the SNAT task (3 runs in total). An additional seven participants were excluded due to data export failures. At W1, 385 participants were included in the MRI analyses (mean age 7.99 ± 0.68, 47% boys, see also [s2]). At W2 48 participants did not start the scan due to anxiety (n=26), contraindications (n=10), or due to lack of parental consent for MRI participation (n=10). 46 participants were excluded at W2 due to excessive head motion and two participants were excluded due to data export failures. At W2 360 participants were included in the MRI analyses (mean age 10.01 ± 0.67, 48% boys).

Of the initial sample that participated at W1, 246 families were contacted 1.5 year after W1 to inform them on a parenting support program for parents of twins (VIPP-Twins [s3]). 91 families (37%) were assigned to the parental intervention group and received the VIPP-Twins, of which 9 families (9.9%) dropped out before the second MRI visit (final VIPP-Twins group: n=164, of which n=133 with sufficient quality MRI (Figure S1)). 129 families (52%) were assigned to the control group and received a dummy intervention, of which 7 families (5.5%) dropped out before the second MRI visit (final control group: n=244, of which n=186 with sufficient quality MRI (Figure S1)). Twenty-seven (11%) families did not want to be randomly assigned to one of the

conditions. These families received the (non-randomly assigned) dummy intervention in order to keep this group comparable to the control group for future analyses within the longitudinal L-CID study. These participants were used as a reference group for regions of interest (ROI) selection. Of the 27 families in the reference group, 3 dropped out before W2. Of the remaining 48 children (Figure S1), 43 participated in the MRI session. Two participants were excluded due to excessive head motion. The final reference group therefore consisted of 41 participants, with a mean age of 10.13 ± 0.71 (age range: 9.09-11.28, 63% boys).

Whole brain analyses reference group

Regions of interest were based on second level group analyses of W2 in a separate reference group (n=41, Table S1). A full-factorial ANOVA with three levels (positive, negative and neutral feedback) was used to investigate the neural response to social feedback in the reference group. Results were Family Wise Error (FWE) cluster corrected (p_{FWEcc}<.05), with a primary voxel-wise threshold of *p*<.005 (uncorrected) [s4]. We first investigated social feedback (positive, neutral, negative) versus fixation. This contrast resulted in activation in amongst others the fusiform gyrus, the inferior frontal gyrus, and the superior frontal gyrus (see Figure S3a and Table S1). In addition to the *social feedback vs fixation* contrast, we also investigated the specific conditions. The contrast *Positive vs Negative feedback* resulted in activation in the right lingual gyrus, the left middle frontal gyrus, and the right inferior parietal lobule (see Table S1, Figure S3b). The contrasts *negative vs positive social feedback* did not result in clusters of significant activation. The contrasts *positive vs neutral social feedback*; and *negative vs neutral social feedback* resulted in increased activation in occipital (visual) cortex (Table S1). All unthresholded statistical maps of the whole brain analyses in the reference group are available through the NeuroVault [S5] repository under: https://identifiers.org/neurovault.collection:6070 (images 306495 – 306499).

Sensitivity analyses on a genetically independent sample

As both the ROI selection (n=41) as well as the analysis including all available MRI data at wave 2 (n=360) are based on twin samples, our results might be influenced by the nestedness of our data. We therefore conducted additional sensitivity analyses on a genetically independent sample. Of the 360 participants with available MRI data at

wave 2 there were 42 “single” twins (i.e., their twin brother/sister was excluded due to motion) and 159 complete twin pairs. Of these complete pairs we randomly selected either the oldest or youngest twin, resulting in a genetically independent sample of n=201. All results were Family Wise Error (FWE) cluster corrected (p_{FWEcc}<.05), with a primary voxel-wise threshold of *p*<.005 (uncorrected) [s4].

A full-factorial ANOVA with three levels (positive, negative and neutral feedback) was used to investigate the neural response to social feedback in the genetically independent sample. We first investigated social feedback (positive, neutral, negative) versus fixation. Similar to the findings of the reference group (Figure S2a), this contrast resulted in activation in several regions including the fusiform gyrus, the bilateral VLPFC and the superior frontal gyrus (see Figure S3a and Table S2). Figure S5a shows the overlap in activation between the genetically independent sample and the regions of interest that were based on the reference group. As can be seen in Table S3, the AI and VLPFC overlapped 100%, the DMPFC overlapped with 84%. Similarly to the reference sample (Figure S3b), the contrast *Positive vs Negative feedback* resulted in activation in the right lingual gyrus, the bilateral DLPFC, and the left superior frontal gyrus (see Figure S4b, Table S2). The overlap for the left DLPFC region was 35% and is visualized in Figure S5b. Moreover, in this sample of n=201, the contrast *Negative vs Positive feedback* resulted in increased activation in the DMPFC, the left VLPFC, and occipital cortex (see Figure S4c, Table S2). The contrasts *negative vs neutral social feedback* resulted in a similar pattern, with increased activation in the DMPFC, the left orbitofrontal cortex, and the occipital cortex (Table S2). Similar to findings in the reference group, the contrast *positive vs neutral social feedback* resulted in increased activation in occipital (visual) cortex (Table S2). All unthresholded statistical maps of the whole brain analyses in the genetically independent group are available through NeuroVault [S5] under: https://identifiers.org/neurovault.collection:6070 (images 306502-306506).

In addition to neural responses to social feedback, we also examined whole brain-behavior relations in the genetically independent sample. We conducted a whole brain regression analysis at the moment of receiving negative social feedback (*negative vs neutral*), with the difference in noise blast duration after negative and neutral feedback as a regressor. Consistent with the results of the complete sample (n=360), we observed a negative association between behavioral aggression and activation in the DLPFC (Table S4). In contrast to the bilateral findings in the larger

complete sample, however, we only observed significant activation in the right DLPFC for the genetically independent sample. As the genetically independent sample is considerably smaller than the complete sample (n=201 versus n=360) this might be due to lower statistical power. Indeed, the unthresholded statistical map (available through NeuroVault [S5]) shows a similar activation pattern in the left DLPFC in the genetically independent sample (https://identifiers.org/neurovault.image:306501) as in the larger whole sample (https://identifiers.org/neurovault.image:306500). The overlap for the significant brain-behavior clusters of the whole sample and the genetically independent sample is visualized in Figure S6.

Supplementary figures

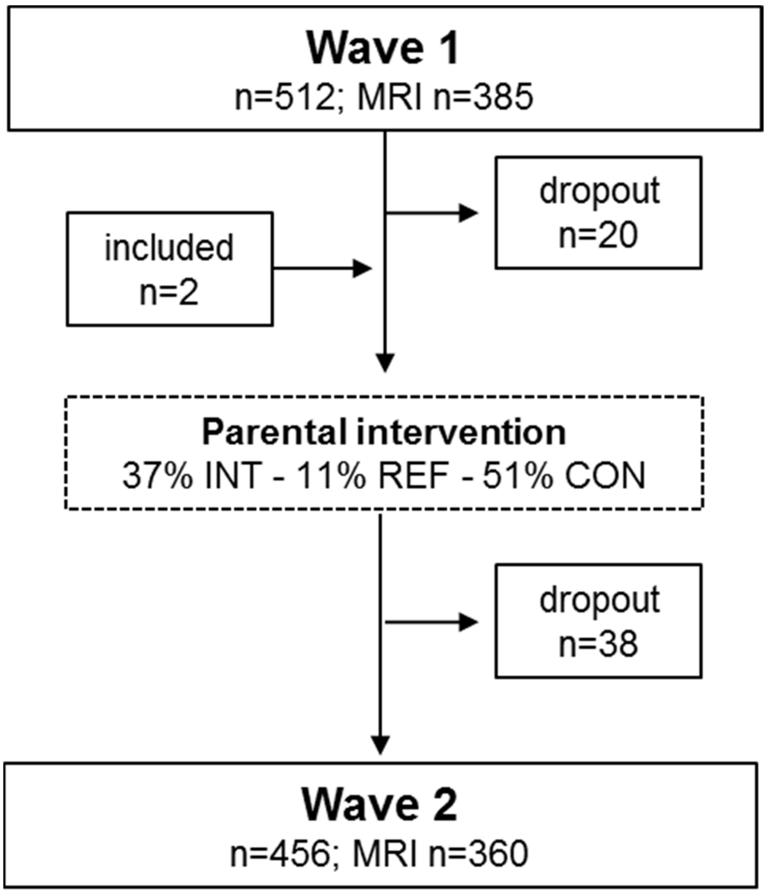

Figure S1. Participant flowchart. INT= intervention group, REF= reference group, CON= control group.

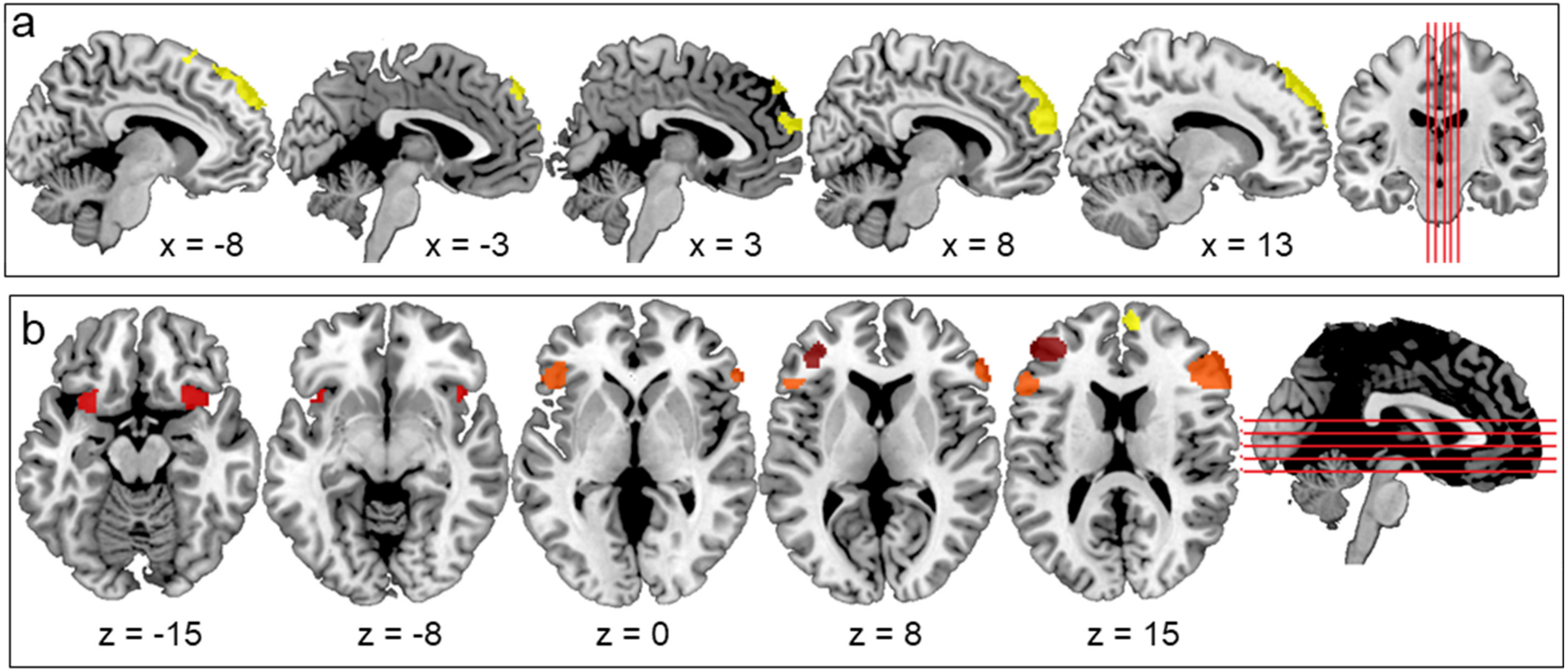

Figure S2. Detailed sagittal (a) and axial (b) multi-slice visualization of the regions of interest. The dorsomedial prefrontal cortex (DMPFC) is displayed in yellow, the anterior insula (AI) in bright red; the ventrolateral prefrontal cortex (VLPFC) in orange and the dorsolateral prefrontal cortex (DLPFC) in dark red. Color coding matches to the render ROI image in the manuscript (Figure 2). Three-dimensional nifti files of the ROIs are accessible through the Open Science Framework (OSF, https://osf.io/a4mdw/).

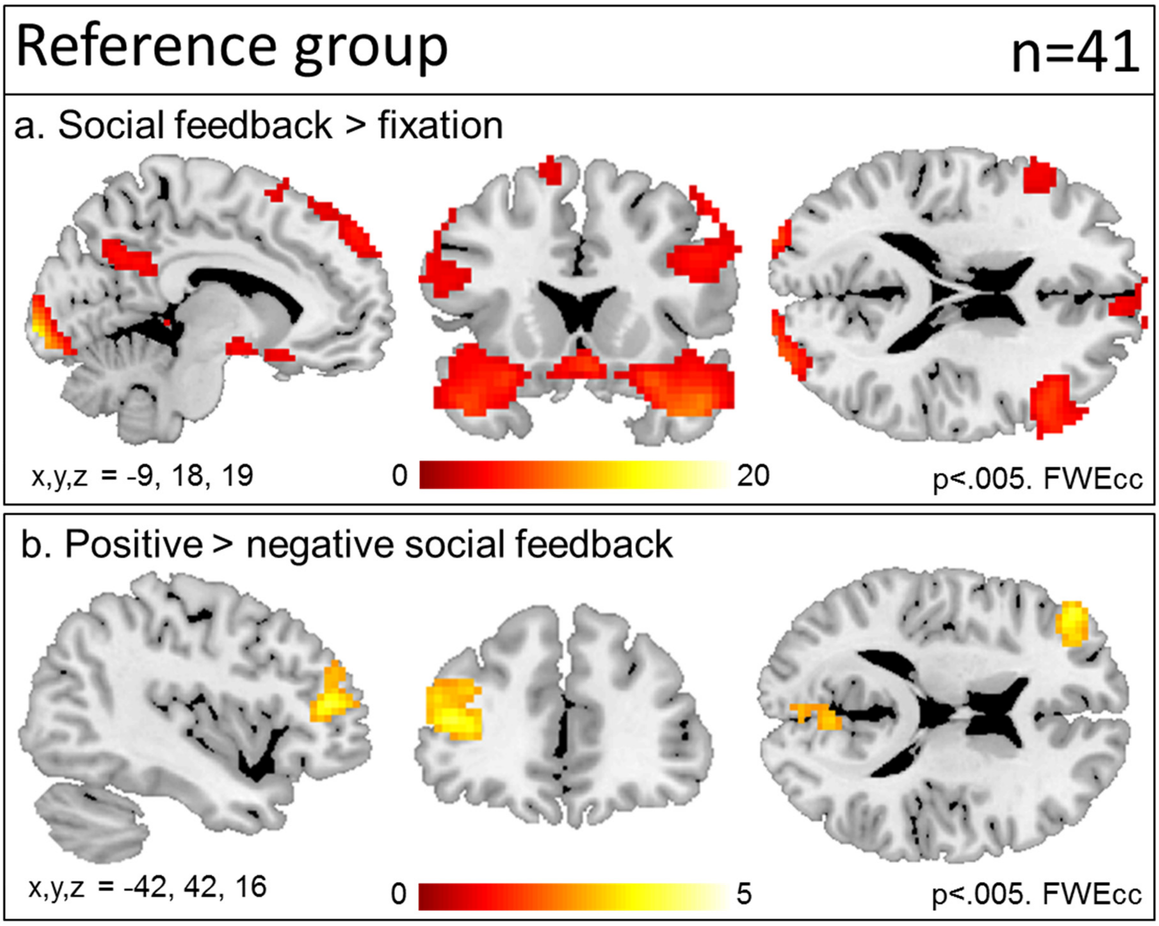

Figure S3. Whole brain analyses for the reference group (n=41).

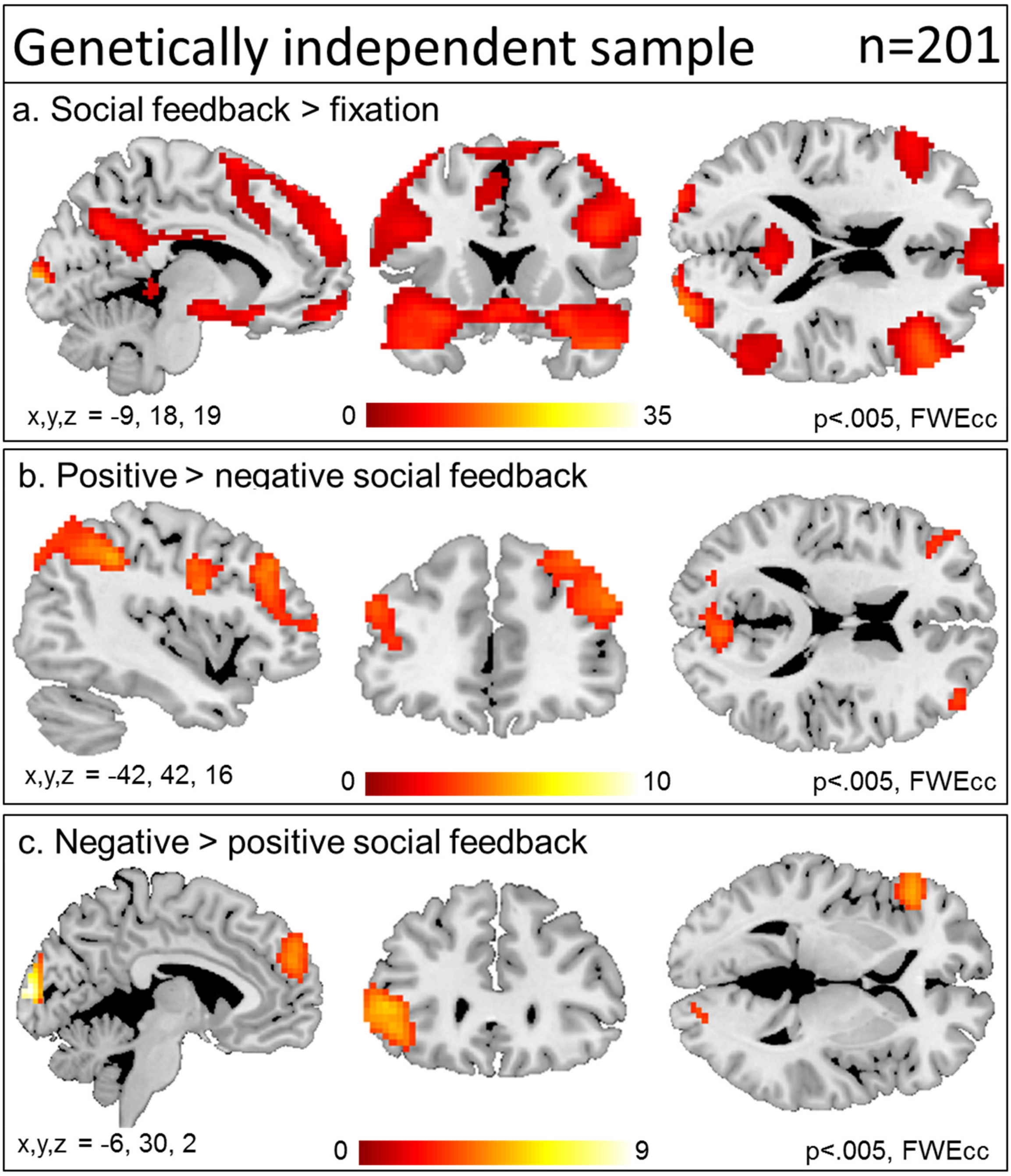

Figure S4. Whole brain analyses in a genetically independent sample (n=201).

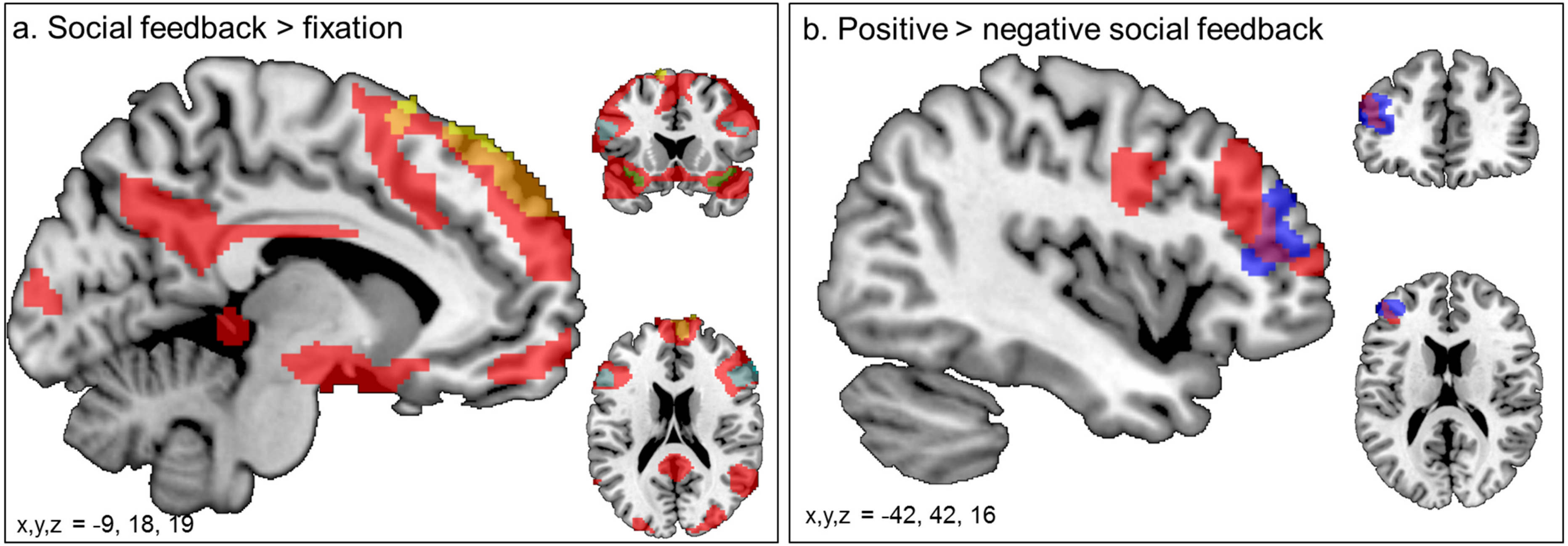

Figure S5. Overlap in neural activation of the genetically independent sample (in red) and the ROIs that were selected based on the reference group. A) ROIs from the social feedback versus fixation contrast, in yellow the dorsomedial prefrontal cortex (DMPFC), in light blue the ventrolateral prefrontal cortex (VLPFC), and in green the anterior insula (AI). B) the left dorsolateral prefrontal cortex (DLPFC) in dark blue.

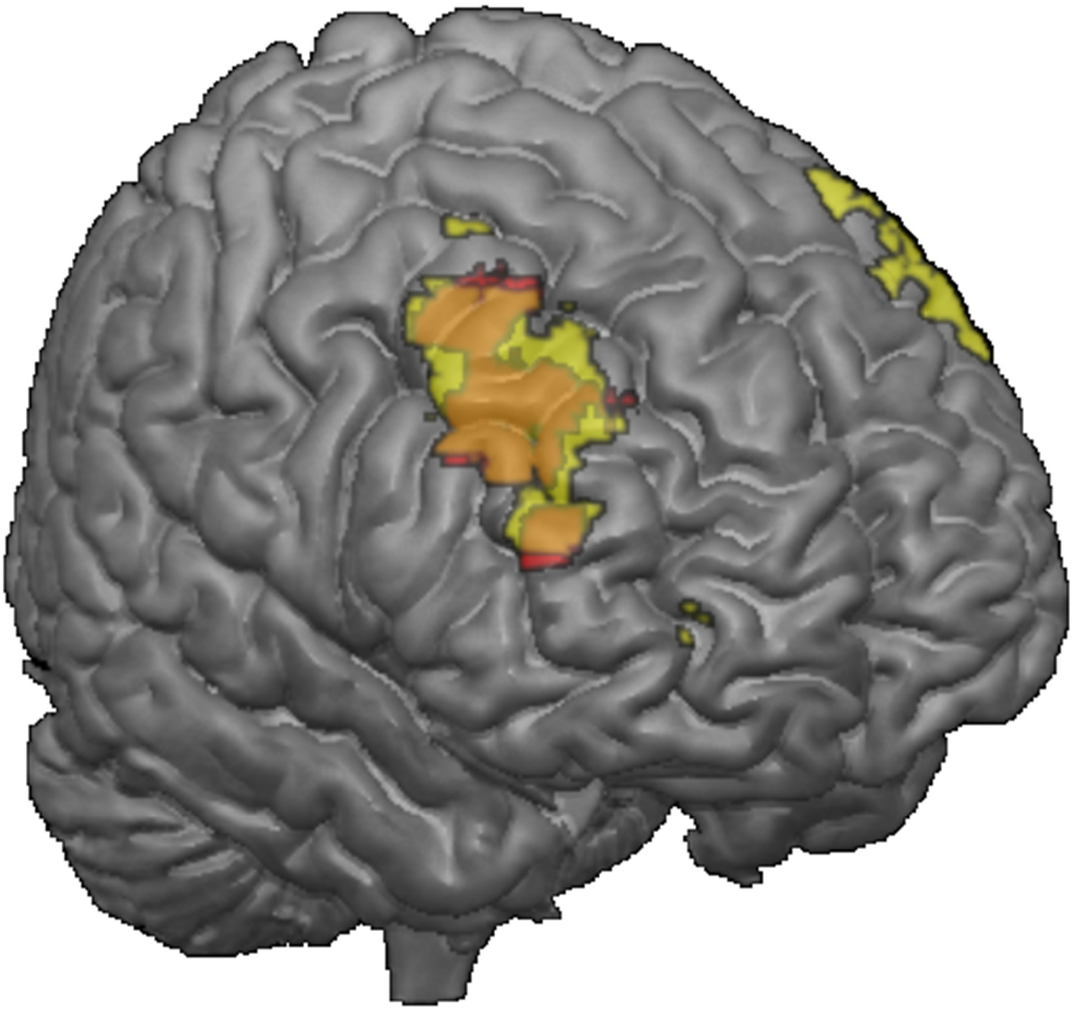

Figure S6. Overlap in neural activation of the brain-behavior association of the complete sample at wave 2 (n=360, in yellow) and the genetically independent sample (n=201, in red).

Supplementary Tables

Table S1. MNI coordinates for local maxima activated for the whole-brain contrasts in the reference group (n=41). Results were FWE cluster corrected (p_{FWEcc}<.05), with a primary voxel-wise threshold of *p*<.005.

Anatomical Region	Voxels	p_{FWEcc}	T	x	y	z
Social feedback > fixation						
Right Fusiform Gyrus	7710	<.001	19.07	39	-52	-17
			18.87	39	-79	-11
			18.61	30	-94	4
Right Posterior Cingulate Cortex	790	<.001	6.53	3	-55	31
			5.03	39	-67	61
			4.87	36	-61	43
Right Inferior Frontal Gyrus	542	<.001	6.11	54	26	22
			6.08	60	29	28
			5.94	45	29	19
Right Superior Frontal Gyrus	453	<.001	4.87	15	50	49
			4.69	-9	53	46
			4.28	-12	38	55
Positive > negative social feedback						
Right Lingual Gyrus	908	<.001	5.43	6	-76	-2
			5.25	-18	-85	-8
			4.70	15	-73	-5
Left Inferior/Middle Frontal Gyrus	185	.040	4.08	-42	41	13
			4.06	-36	47	13
			3.26	-39	44	25
Positive > neutral social feedback						
Left Fusiform Gyrus	3186	<.001	6.43	-27	-79	-11
			6.41	24	-70	-11
			5.98	12	-76	-8
Negative > neutral social feedback						
Left Middle Occipital Gyrus	1958	<.001	7.05	-48	-79	4
			6.10	-12	-97	16
			5.29	45	-82	7

Table S2. MNI coordinates for local maxima activated for the whole-brain contrasts in the genetically independent sample (n=201). Results were FWE cluster corrected (p_{FWEcc}<.05), with a primary voxel-wise threshold of *p*<.005.

Anatomical Region	Voxels	p_{FWEcc}	T	x	y	z
Social feedback > fixation						
Right Fusiform Gyrus	15845	<.001	36.33	39	-46	-20
			33.73	30	-91	4
			33.26	21	-97	7
Positive > negative social feedback						
Right Lingual Gyrus	2319	<.001	10.26	6	-73	1
			7.52	21	-70	-5
			7.34	-24	-76	-5
Right Dorsolateral Prefrontal Cortex	1891	<.001	6.18	39	35	46
			5.39	6	23	46
			5.37	27	8	58
Right Lateral Occipipital Cortex	899	<.001	5.52	45	-73	43
			5.40	57	-40	52
			5.01	51	-37	46
Left Superior Frontal Gyrus	282	.019	5.35	-27	11	58
			3.22	-24	-1	46
Left Dorsolateral Prefrontal Cortex	463	.001	4.43	-42	35	37
			4.39	-48	5	34
			4.29	-45	35	28
Negative > positive social feedback						
Bilateral Occipital Pole	449	.002	9.39	-6	-97	10
			8.63	-12	-94	19
			5.53	24	-91	16
Left Ventrolateral Prefrontal Cortex	471	.001	5.62	-54	26	7
			4.92	-48	29	-2
			4.58	-42	23	-11
Dorsomedial Prefrontal Cortex	324	.010	4.81	-9	59	37
			4.42	-6	53	28
			4.40	-21	53	34

Table S2. *(Continued)*

Anatomical Region	Voxels	p_{FWEcc}	T	x	y	z
Positive > neutral social feedback						
Right Occipital Fusiform Gyrus	4307	<.001	11.27	24	-70	-8
			10.4	-27	-76	-5
			9.52	5	-73	-2
Negative > neutral social feedback						
Left Lateral Occipital Cortex		<.001	13.57	-48	-82	4
			11.23	-6	-97	10
			10.81	-12	-94	19
Left Orbitofrontal Cortex		.002	6.19	-42	26	-8
			3.58	-30	32	4
			3.40	-36	8	-2
Dorsomedial Prefrontal Cortex		.027	5.06	-6	56	25
			4.69	9	65	34
			3.73	-9	62	37

Table S3. Number of voxels in region of interest (ROI) based on the reference group and the number and percentage of overlap with significant whole brain activation in the same contrast using a larger, genetically independent sample (n=201).

ROI	Based on whole brain contrast	voxels	overlap° voxels	overlap° percentage
AI (bilateral)	Social feedback *vs* fixation	401	401	100%
DMPFC	Social feedback *vs* fixation	1442	1217	84%
VLPFC (bilateral)	Social feedback *vs* fixation	1465	1438	98%
DLPFC (left)	Positive *vs* negative feedback	598	212	35%

° *compared with a genetically independent sample of n=201*

Table S4. MNI coordinates for local maxima activated for the whole brain-behavior contrast in the genetically independent sample (N=201). Results were FWE cluster corrected (p_{FWEcc}<.05), with a primary voxel-wise threshold of *p*<.005.

Anatomical Region	Voxels	p_{FWEcc}	T	x	y	z
Negative feedback > Neutral feedback, with Noise blast regressor (Δ Negative - Neutral)						
Right dorsolateral prefrontal cortex	260	.023	3.84	48	32	40
			3.53	42	23	52
			3.17	45	44	19

Table S5. Intra class coefficients between wave 1 and wave 2 brain activation in region of interest. AI: anterior insula; DMPFC: dorsomedial prefrontal cortex; VLPFC: ventrolateral prefrontal cortex; DLPFC: dorsolateral prefrontal cortex; CI= confidence interval

ROI	contrast	ICC	95% CI lower bound	95% CI upper bound
AI	negative > positive	-0.05	-0.16	0.07
(bilateral)	negative > neutral	0.05	-0.07	0.16
	positive > neutral	-0.03	-0.14	0.09
DMPFC	negative > positive	-0.08	-0.20	0.03
	negative > neutral	0.06	-0.05	0.17
	positive > neutral	0.03	-0.09	0.14
VLPFC	negative > positive	-0.05	-0.16	0.07
(bilateral)	negative > neutral	0.10	-0.02	0.21
	positive > neutral	0.05	-0.06	0.17
DLPFC	negative > positive	0.04	-0.08	0.15
(left)	negative > neutral	0.04	-0.07	0.16
	positive > neutral	0.05	-0.06	0.16

Table S6. Linear mixed effect model with noise blast duration as dependent variable. Output is based on type III ANOVA's using Satterthwaite's method. Significant effects are depicted in black fonts, insignificant effects in grey.

**Linear Mixed Effect Models°**	***DF***	***F***	***p***
Condition	2, 2181.60	1033.61	<0.001
Wave	1, 2185.79	157.17	<0.001
Gender	1, 217.86	1.21	0.273
Estimated IQ	1, 406.16	0.01	0.928
Intervention Group	1, 217.81	0.07	0.795
Conditon × Wave	2, 2181.60	16.06	<0.001
Conditon × Gender	2, 2181.60	2.26	0.104
Conditon × Estimated IQ	2, 2181.60	13.55	<0.001
Conditon × Intervention	2, 2181.60	0.65	0.523
Wave × Intervention	2, 2185.79	3.18	0.075
Conditon × Wave × Intervention	2, 2181.60	0.14	0.874

° ***Results were similar for models with and without IQ as covariate***

Table S7. Linear mixed effect model with brain activation in regions of interest as dependent variable. Output is based on type III ANOVA's using Satterthwaite's method. Significant effects are depicted in black fonts, insignificant effects in grey.

Linear Mixed Effect Models	*DF*	*F*	*p*
Anterior Insula°			
Condition	2, 1526.24	27.79	<0.001
Wave	1, 1783.92	10.09	<0.001
Gender	1, 182.36	0.19	0.663
Estimated IQ	1, 313.02	1.88	0.171
Intervention Group	1, 181.80	0.00	0.953
Conditon × Wave	2, 1526.24	2.06	0.127
Conditon × Gender	2, 1526.24	0.83	0.435
Conditon × Estimated IQ	2, 1526.24	0.61	0.544
Conditon × Intervention	2, 1526.24	0.83	0.437
Wave × Intervention	1, 1783.75	0.11	0.737
Conditon × Wave × Intervention	2, 1526.24	0.93	0.394
Dorsomedial Prefrontal Cortex°			
Condition	2, 1530.08	6.64	0.001
Wave	1, 1790.59	5.61	0.018
Gender	2, 1530.08	0.64	0.527
Estimated IQ	1, 161.60	0.93	0.337
Intervention Group	1, 161.09	0.69	0.408
Conditon × Wave	2, 1530.08	0.61	0.543
Conditon × Gender	2, 1530.08	0.44	0.646
Conditon × Estimated IQ	2, 1530.08	1.32	0.267
Conditon × Intervention	1, 1790.41	2.43	0.119
Wave × Intervention	2, 1530.08	0.26	0.769
Conditon × Wave × Intervention	1, 314.32	0.03	0.853
Ventrolateral Prefrontal Cortex°			
Condition	2, 1531.45	8.22	<0.001
Wave	1, 1804.24	0.54	0.461
Gender	1, 175.66	2.53	0.113
Estimated IQ	1, 278.98	0.52	0.471
Intervention Group	1, 175.23	0.15	0.696
Conditon × Wave	2, 1531.45	1.58	0.205
Conditon × Gender	2, 1531.45	2.10	0.123
Conditon × Estimated IQ	2, 1531.45	0.84	0.430
Conditon × Intervention	2, 1531.45	0.60	0.549
Wave × Intervention	1, 1804.11	3.17	0.075
Conditon × Wave × Intervention	2, 1531.45	0.00	0.997

° *Results were similar for models with and without IQ as covariate*

Table S7. *(continued)*

Linear Mixed Effect Models	*DF*	*F*	*p*
Dorsolateral Prefrontal Cortex°			
Condition	2, 1532.09	8.21	0.000
Wave	1, 1788.11	34.44	0.000
Gender	1, 188.49	0.05	0.827
Estimated IQ	1, 300.71	5.67	0.018
Intervention Group	1, 187.98	0.00	0.993
Conditon × Wave	2, 1532.09	2.53	0.080
Conditon × Intervention	2, 1532.09	0.95	0.386
Conditon × Gender	2, 1532.09	1.98	0.138
Conditon × Estimated IQ	2, 1532.09	4.21	0.015
Wave × Intervention	1, 1787.97	0.10	0.747
Conditon × Wave × Intervention	2, 1532.09	0.04	0.958

° Results were similar for models with and without IQ as covariate

Table S8. Linear mixed effect models with brain activation in regions of interest as dependent variable and noise blast duration added as factor. Output is based on type III ANOVA's using Satterthwaite's method. Significant effects are depicted in black fonts, insignificant effects in grey.

Linear Mixed Effect Models	*DF*	*F*	*p*
Anterior Insula°			
Condition	2, 1693.46	14.59	<0.001
Wave	1, 1808.94	9.26	0.002
Noise blast	1, 1908.4	5.47	0.019
Gender	1, 182.85	0.29	0.592
Estimated IQ°	1, 317.03	1.91	0.168
Intervention Group	1, 181.67	0.01	0.907
Conditon × Wave	2, 1659.61	1.18	0.306
Conditon × Noise blast	2, 1728.48	1.09	0.337
Conditon × Gender	2, 1526.86	0.90	0.406
Conditon × Estimated IQ	2, 1531.67	0.56	0.569
Conditon × Intervention	2, 1525.39	0.81	0.447
Wave × Noise blast	1, 1913.40	1.74	0.188
Wave × Intervention	1, 1785.34	0.13	0.718
Conditon × Wave × Noise blast	2, 1676.93	0.45	0.637
Conditon × Wave × Intervention	2, 1525.17	1.03	0.356
Dorsomedial Prefrontal Cortex°			
Condition	2, 1699.33	5.73	0.003
Wave	1, 1816.12	8.20	0.004
Noise blast	1, 1895.96	2.71	0.100
Gender	1, 160.39	1.17	0.282
Estimated IQ°	1, 316.05	0.00	0.959
Intervention Group	1, 159.34	0.81	0.370
Conditon × Wave	2, 1665.49	1.00	0.369
Conditon × Noise blast	2, 1736.36	0.48	0.621
Conditon × Gender	2, 1531.15	1.39	0.248
Conditon × Estimated IQ	2, 1536.06	0.39	0.675
Conditon × Intervention	2, 1529.67	0.52	0.594
Wave × Noise blast	1, 1910.81	4.59	0.032
Wave × Intervention	1, 1791.29	2.22	0.136
Conditon × Wave × Noise blast	2, 1684.20	1.31	0.271
Conditon × Wave × Intervention	2, 1529.44	0.20	0.815

° Results were similar for models with and without IQ as covariate

Table S8. *(continued)*

**Linear Mixed Effect Models**		***F***	***p***
Ventrolateral Prefrontal Cortex°			
Condition	2, 1709.10	5.45	0.004
Wave	1, 1830.86	0.83	0.363
Noise blast	1, 1872.52	2.57	0.109
Gender	1, 174.04	2.89	0.091
Estimated IQ°	1, 280.10	0.80	0.373
Intervention Group	1, 172.89	0.20	0.655
Conditon × Wave	2, 1673.03	1.60	0.202
Conditon × Noise blast	2, 1752.47	0.34	0.711
Conditon × Gender	2, 1528.65	2.22	0.109
Conditon × Estimated IQ	2, 1533.92	0.70	0.498
Conditon × Intervention	2, 1527.06	0.52	0.592
Wave × Noise blast	1, 1929.34	1.07	0.302
Wave × Intervention	1, 1804.98	3.14	0.077
Conditon × Wave × Noise blast	2, 1695.95	0.06	
Conditon × Wave × Intervention	2, 1526.81	0.01	0.994
Dorsolateral Prefrontal Cortex*			
Condition	2, 1697.56	0.93	0.396
Wave	1, 1810.80	10.22	0.001
Noise blast	1, 1911.70	4.32	0.038
Gender	1, 188.65	0.01	0.912
Estimated IQ *	1, 305.09	5.19	0.023
Intervention Group	1, 187.45	0.00	0.958
Conditon × Wave	2, 1664.25	3.34	0.036
Conditon × Noise blast	2, 1731.91	0.70	0.499
Conditon × Gender	2, 1533.61	1.96	0.141
Conditon × Estimated IQ	2, 1538.32	4.88	0.008
Conditon × Intervention	2, 1532.15	1.08	0.339
Wave × Noise blast	1, 1918.14	0.07	0.797
Wave × Intervention	1, 1788.91	0.12	0.726
Conditon × Wave × Noise blast	2, 1680.74	1.48	0.228
Conditon × Wave × Intervention	2, 1531.93	0.03	0.968

° Results were similar for models with and without IQ as covariate
** After excluding IQ as covariate the effects of Noise blast (F=3.77, p=.052) and Condition × Wave (F=2.96, p=.052) were no longer significant*

Table S9. Behavioral genetic modelling of behavioral aggression related DLPFC activation at wave 1, wave 2, and change across time (residualized change scores).

		MZ	DZ	A	C	E
DLPFC wave 1	*r*	.05	.07	0.00	0.06	0.94
	n	87	71	[± - 0.27]	[0.00 - 0.23]	[0.77 - ±]
DLPFC wave 2	*r*	.11	.14	0.00	0.15	0.85
	n	86	72	[± - 0.33]	[0.00 - 0.32]	[0.68 - ±]
DLPFC change	*r*	.03	.16	0.00	0.09	0.91
	n	66	48	[± - 0.27]	[0.00 - 0.28]	[0.71 - ±]

± *confidence intervals could not be estimated*

Supplementary references

S1 Achterberg, M. & van der Meulen, M. Genetic and environmental influences on MRI scan quantity and quality. *Developmental cognitive neuroscience* **38**, 100667, doi:10.1016/j.dcn.2019.100667 (2019).

S2 Achterberg, M., van Duijvenvoorde, A. C. K., van der Meulen, M., Bakermans-Kranenburg, M. J. & Crone, E. A. Heritability of aggression following social evaluation in middle childhood: An fMRI study. *Human brain mapping* **39**, 2828-2841, doi:10.1002/hbm.24043 (2018).

S3 Euser, S. *et al.* Efficacy of the Video-feedback Intervention to promote Positive Parenting and Sensitive Discipline in Twin Families (VIPP-Twins): Study protocol for a randomized controlled trial. *BMC Psychol* **4**, 33, doi:10.1186/s40359-016-0139-y (2016).

S4 Woo, C. W., Krishnan, A. & Wager, T. D. Cluster-extent based thresholding in fMRI analyses: Pitfalls and recommendations. *NeuroImage* **91**, 412-419, doi:10.1016/j.neuroimage.2013.12.058 (2014).

S5 Gorgolewski, K. J. *et al.* NeuroVault.org: a web-based repository for collecting and sharing unthresholded statistical maps of the human brain. *Front Neuroinform* **9**, 8, doi:10.3389/fninf.2015.00008 (2015).
